# The Oddity of Heterogeneity: A Blessing in Disguise

**DOI:** 10.1038/s41598-018-29081-7

**Published:** 2018-07-17

**Authors:** Yuhui Lin

**Affiliations:** The Waterhouse at NaoRococo, Singapore, Singapore

## Abstract

Damage accumulation is widely accepted as the central dogma of ageing, and it has been a long-standing belief that tobacco smokers must experience a faster rate of ageing than non-smokers. It is therefore puzzling as to why proportional hazard model is a popular choice in longitudinal studies given that its assumption assumes a constant hazard with increasing time. If the rate of ageing is accelerated, the hazard gradient of smokers *d(log(μ(x)))/dx* obtained from frailty parametric fit has to be steeper than non-smokers. This study examines the relative derivative for mortality *d(log(μ(x)))/dx* of British doctors born 1900–1909, and obtained estimates indicate that the rate of ageing is similar between smokers and non-smokers. A brief theorem is also elaborated to present the difference in life-years gained from interventions and policies by life-detrimental risk exposure; *e*.*g*. smokers 0.8; non-smokers 5.3 mins/day. The controversial assumption made in the central dogma of ageing, heterogeneity axiom and the application of proportional hazard models are unveiled in this condensed parametric analyses.

## Introduction

Many previous studies have shown that tobacco smoking is associated with an increased risk for chronic health diseases such as breast cancer, myocardial infarction and dementia^1–3^. Elevated risk for mortality among tobacco smokers indicates that tobacco smoking is a persistent life-detrimental behaviour across all socioeconomic groups, genders, occupations and countries^4–7^. Therefore, there has not been a fundamental reason to raise the hypothesis that the rate of ageing among smokers is not accelerated. This central dogma however contradicts the popular statistical choice for proportional hazard models in longitudinal studies, specifically in prospective studies. If damage accumulation from tobacco smoking accelerates the rate of ageing and increases the risk for mortality, the relative risk for mortality between smokers and non-smokers will diverge with increasing age. Proportional hazard models which assume a constant hazard risk with increasing age will therefore be void, *i*.*e*. a constant hazard is equivalent to parallel hazard lines between *log μ(x)*_exposed_ and *log μ(x)*_non-exposed_ group with increasing age.

## Results and Discussion

### Examining risk and rate from a mixed bag of choices

To examine whether tobacco smokers experience (I) a higher magnitude for mortality risk *log μ(x)*; (II) a faster rate of ageing *d(log(μ(x)))/dx*; (III) both mortality risk and rate of ageing are elevated to non-smokers; the age-specific mortality trajectory of British doctors born in year 1900–1909 is presented by fitting a parametric model to determine the shape and pace of the hazard of smokers and non-smokers; Supplementary Table S1. The rate of ageing is determined by the relative derivative for mortality; *d(log(μ(x)))/dx*, best known as the slope of the hazard line on semi-logarithmic plot. The faster the rate of ageing, the steeper the hazard slope.

In human mortality studies, it is an analytical ritual to fit a Gompertz hazard function to mortality data between ages 30 and 90^8^. In the event whereby excess non-senescence related deaths were to occur during young adulthood, a Makeham term ‘*c*’ is included to the Gompertz function; Gompertz-Makeham^9^. In conjunction to the usual Gompertz function, heterogeneity was also taken into consideration during the parametric analysis; Equation 1 and Equation 2. Aside from prenatal genetics and epigenetic exposures, individuals born in the same cohort will have to undergo selection for mortality since day one of survival^10^. Heterogeneity occurs when individuals are given the choice to choose their dietary habits and to experience communicable diseases in their environment. The combination of behaviours driven by individuals’ preference or by force to reside in a confined environment creates different magnitude for selection of death; *log μ(x)*. This further illustrates that if unobserved heterogeneity or covariates (*i*.*e*. observed heterogeneity) are not accounted for during survival or life-table analyses, the obtained risk estimates will be misleading^11^.

#### Equation 1. Individual hazard *h(x)* assuming a Gompertz-Makeham baseline

Frailty indicator is represented as *Z* which is a random positive integer; *Z* > 0. Frail individuals will have higher *Z*-values than robust individuals.

$$h(x)=Z.a{e}^{bx}+c$$

#### Equation 2. Population mortality rate *μ(x)* with a gamma distributed frailty, *Z(x)*

which is also known as the variance. Parameters: *a* is the magnitude of mortality risk in the Gompertz function, *b* represents demographic ageing which defines the gradient of the hazard line, *c* accounts for non-senescence deaths and Ɣ is the frailty parameter for unobserved heterogeneity; *x* as ages at death.

$${\rm{\mu }}({x})=\frac{a{e}^{bx}}{Z(x)}+c=\frac{a{e}^{bx}}{1+\frac{a\gamma }{b}({e}^{bx}-1)}+c$$

The next controversial analytical perspective lies in heterogeneity. If frail individuals are selected to experience mortality earlier than robust individuals, frail smokers will experience death earlier than non-smokers and robust smokers. The selection for mortality shall hereafter be termed as frailty. Therefore in a heterogeneous population, selection for mortality from tobacco smoking would occur from the beginning of survival time to the end of observation time. Smokers *log(μ(x))* will have to converge to non-smokers *log(μ(x))* with increasing age, and the explanation is that frail smokers are selected to expire at a much faster pace than non-smokers at young ages. At high ages, robust long-term smokers would then be considered as the ‘lucky ones’ to remain in the population and to experience a similar hazard than of non-smokers. This aforementioned statement which is based on the fundamentals of natural selection should require no subtlety concerning heterogeneity by tobacco smoking, but it conflicts with the central dogma of accelerated ageing by smoking behaviours.

**Figure 1:**
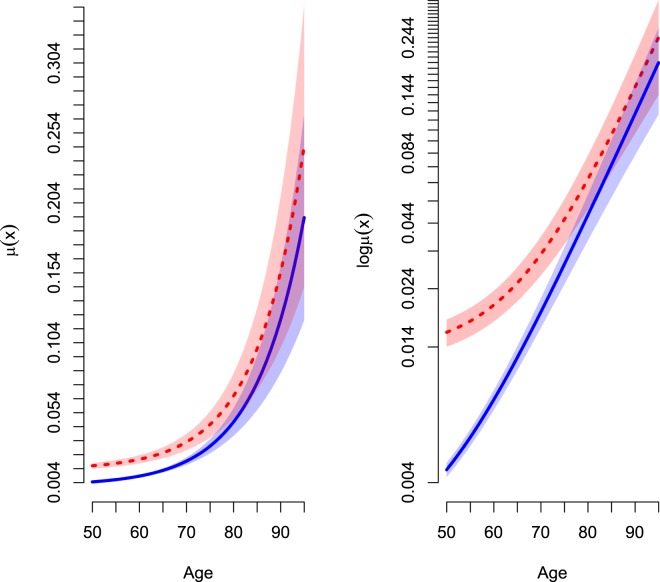
Age-specific mortality trajectories obtained from Gompertz-Makeham Frailty analyses. Smokers (Dotted); Non-smokers (Solid). Absolute (left); Semi-logarithmic scale (right). Mortality rates were not manipulated, and were expressed on a different scale to present a simple mathematical transformation to unveil mortality dynamics and interpretation of the parameter estimates.

Figure 1 shows the hazard lines of smokers and non-smokers on the absolute scale and semi-logarithmic scale. The non-nested parametric hazard lines and its relative risk for mortality between smokers and non-smokers indicate a converging trend with increasing age. This finding neither accepts the central dogma of ageing whereby accelerated ageing must occur among tobacco smokers nor the parallel lines assumption made in proportional hazard model. Hence, it is true that individuals born in the same birth cohort and when a life-detrimental risk exposure is examined with the aid of age-specific mortality trajectory; *log μ(x) not log μ(t)*, convergence will occur if individuals are able to live long enough and to be engaged to the habit for a long-term before the onset of life-threatening diseases; *e*.*g*. more than 40 years of smoking habit.

Before 1960s in the UK and Europe, tobacco smoking was considered as a socially acceptable behaviour as there was little evidence to justify its harmful effects on longevity and health^12^. The 1900–1909 decennial cohort received no or little health intervention, and smokers who quit their long-term smoking habits were likely to be diagnosed for chronic diseases and were too frail to smoke. These smokers were likely to experience death within one to two years of clinical diagnosis^13^.

### Defining heterogeneity axiom from standard individual mortality trajectories

**Figure 2:**
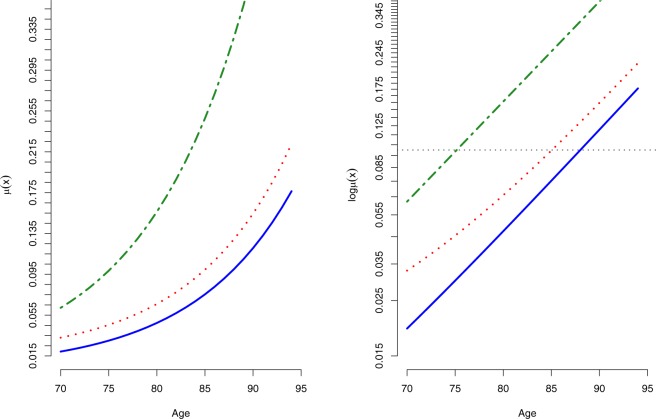
Standard individual age-specific mortality trajectories. Adjusted mortality rates for heterogeneity both observed and unobserved given availability of data. General Population (Alternate dashed-dotted); Smokers (Dotted); Non-smokers (Solid). Horizontal line indicates mortality at 10%; a decrement in three years of life expectancy between smokers and non-smokers.

Though the survival curves published in the British Doctors’ study were already adjusted for heterogeneity, the general population was presented in this analysis to justify that when no covariate is recorded or made available for statistical adjustment, a Gompertz-Makeham function with a gamma distributed frailty is capable to account for unobserved heterogeneity in mortality schedules^14,15^; Supplementary Tables S2 and S3. In addition, the general population served as a useful representation of standard individual ‘control’ group of the adjusted risk and parameter estimates, and the validation of proportional hazard models for comparative analyses in tobacco studies when all heterogeneity have been accounted for. By fitting a frailty Gompertz-Makeham model to both British Doctors and general population, it would then be feasible and realistic to compare the adjusted risk and rate estimates; Fig. 2: Standard individual mortality trajectories. When mortality is at 10%, smokers experienced a three years decrement in life-expectancy to non-smokers. The Makeham-term ‘*c*’ which is often used in demographic studies to describe non-senescence deaths driven by reckless behaviours such as road accidents has shown to be significantly different between smokers and non-smokers, Table S2 and a pronounced mortality ‘hump’ for the smokers hazard line Figs 1 and 2. My finding suggests that medical doctors who were born in the early 1900s were likely to have shared very similar socio-economic status resulting to a similar ‘*a*’ parameter in the parametric-frailty analysis, but their additional risk for mortality was attributed from risk-taking behaviours that were associated with tobacco smoking including binge drinking and speeding on roads.

Based on the heterogeneity axiom, it is within expectation that the adjusted age-specific mortality trajectory of smokers, non-smokers and the general population present three parallel lines.

### The dilemma when Gompertz hazard is sufficient

To test the accelerated failure time hypothesis, a Weibull model or AFT functions should be fitted to the mortality schedules by smoking behaviours. However, the raw mortality estimates presented a Gompertzian baseline hazard shape of smokers, as similar to non-smokers. If Weibull is the most appropriate fit to smokers, regardless of the simplicity of the Gompertzian function, the hazard lines of non-fitted mortality must diverge; Supplementary Fig. S1.

As the findings demonstrate the human rate of ageing does not differ by smoking behaviour, selection for mortality which outlines the frailty distribution is likely to be solely dependent on magnitude for mortality risk than our previously presumed central dogma - the rate of ageing. The findings also suggest that despite long-term tobacco smoking exposure on the human ageing rate, the Gompertzian *b* parameter in a normal healthy population or group of individuals appears to be a robust vitality element. As long as the life-detrimental risk exposure does not provoke an immediate death, but rather an accumulation of damage which also offers time for repair or damage-sustained control, the rate of ageing of the standard exposed individual will be similar to non-exposed standard individual.

### Average time gained from health interventions, mins per day

Lastly, a brief theorem of life-years gained from interventions and policies. Britain experienced advancements in mortality reduction since year 1900; Supplementary Fig. S1. This illustration presents whether the accountability of intervention effectiveness of life-saving opportunities against tobacco smoking can be better interpreted using life-years loss or gained from the obtained age-specific mortality trajectories. Based on initial mortality risk of smokers, life-years gained from interventions would be lower among smokers to non-smokers and the deduced estimates were converted to mins per day of life-years gained; Table 1. Medical doctors have the capabilities to save patients’ lives and in comparison to the general population, they are more equipped with the knowledge to recognising early symptoms of disease onset. Among British doctors, non-smokers gained 5.3 mins per day from interventions whereas smokers gained 0.8 mins per day. Though doctors whom smoked had a lower life-years gained than the general population, it is essential to elaborate that the average life-years obtained from males in the general population contained both smokers and non-smokers; 1.3 mins per day. Smokers in the general population would have had experienced <0.8 mins per day of life-years gained.

**Table 1:** Average annual mortality improvement and life-years gained based on age at 70, cohorts 1900–1929; ω_70, 1900–1929_.

	Non smokers, doctors	Smokers, doctors	General Population
ω_70, 1900–1929_	0.370%	0.056%	0.087%
Life-years (mins/day)	5.3 mins	0.8 min	1.3 mins

*N*.*B*: General population mortality rates were obtained from the Human Mortality Database (HMD); minutes as mins.

## Conclusion

Proportional hazard model and its assumption appear to be an appropriate statistical-choice in longitudinal studies concerning tobacco smoking, and it is important that heterogeneous effects both observed and unobserved must be accounted for to ensure that the parallel lines assumption is not violated. The irony in the findings is that the central dogma of ageing is not correct for tobacco smoking-related non-cancer and cancer mortality, and the heterogeneity axiom stands to be true. It is a blessing in disguise that death which occurs once in an individual’s lifetime has not altered the rate of mortality increase per unit of age; referred as the rate of ageing in text; *d(log(μ(x)))/dx*. Smokers do experience a higher risk for mortality, but the rate of ageing remains similar as to that of non-smokers. Proportional hazard model is useful and can be considered as an intelligent statistical approach during survival analysis of tobacco studies^16^. The brief theorem of life-years gained would be a more effective measurement to life-saving opportunities and for interventional reports on costings than mortality risk alone.

## Methods

The data was extracted from the published survival curves of Doll and colleagues (2004)^17^. In this study, the 1900–1909 decennial birth cohort is appropriate for a parametric fit as there were adequate number of deaths at high ages beyond 80 s and at the year of last survival follow-up 2004, the published survival curves implied that it was close to a complete case in both smokers and non-smokers. The power of the study could be increased if no advancement was made on cohort mortality, and cohorts of 1900–1929 could be analysed as a category for a mortality trajectory analysis. However, the progressive mortality reduction as evidently seen in Supplementary Fig. S2 would lead to statistical bias if the three decennial birth cohorts were to be aggregated during statistical analyses. The reduction in mortality rate occurs in continuous time rather than across ages at death as shown in Equation 2; considered as unobserved heterogeneity. This statistical bias remains true in individual survival profiles, and hence a life-table analysis must also consider for advancements made in medicine over calendar time.

As the extracted data was retrieved using survival curves, a Binomial Gompertz hazard function was used to obtain the parameter estimates contributing to its age-specific mortality trajectory. Heterogeneity occurs in a population, and hence a distribution must be present to outline the pace and the scale of the selection process; frail individuals expire quicker than robust or less frail individuals. A gamma-distribution is assumed as the frailty component of the population; each individual is given a Z-value as its frailty indicator; the higher the Z-value the likelihood for death increases, Equation 1. Z-value will always be positive, thereby the given hazard rate of an individual will not be negative; a negative mortality human rate would be nonsensical. N.B: Mortality describes population death; and death represents individual’s event.

The combination of Z-values among individuals in the population contributes to *Z(x)* which is also known as the average frailty. When the model is adjusted for heterogeneity, the standard individual is represented as *Z* = *1*. The frailty model would then return to the classic Gompertz-Makeham framework presenting the standard individual hazard. In order for selection process to occur, the mortality hazard must undergo a division; Equation 2.

The age-specific mortality trajectory of 1900–1909 birth cohort of British male doctors by smoking status was also compared to their respective males in the general population; Data source: Human Mortality Database (HMD)^18^. The average mortality progress was deduced from life-tables^19^. From the percentage deduction, life-years gained from progress made across calendar time was obtained by converting percentage of reduction in mortality to time gained per day.

All estimates were obtained using Maximum Likelihood Estimation and optimized parameters reached convergence. Statistical analyses were conducted using R-software version 3.2.1^20^.

## Electronic supplementary material

Supplementary information
