# Supplementary material for: The Oddity of Heterogeneity: A Blessing in Disguise
Source: Sci Rep. 2018 Jul 17;8:10782. doi: 10.1038/s41598-018-29081-7 (PMC6050328; doi:10.1038/s41598-018-29081-7)

SUPPLEMENTARY INFORMATION

Title of Manuscript The Oddity of Heterogeneity: A Blessing in Disguise

Author Yuhui Lin

Tables

- **Table S1**. Digitized Survivorship Data Extraction from *Doll et. al 2004*
- **Table S2**. British Male Doctors parameter estimates obtained from Maximum Likelihood Estimation.
- **Table S3**. Gompertzian models and parameter estimates for British Male General Population.

Figures

- **Figure S1.** Raw mortality rates from extracted survival curves on the absolute and semi-logarithmic scale.
- **Figure S2**. Mortality rate at age 70 across calendar years.

Table S1. **Digitized Survivorship.** Data Extraction from *Doll et. al 2004* British Doctors 50 years survival follow-up; Figure S6. NS: Non-Smokers; S: Smokers; lx: Proportion of Survivors; μ(x): Mortality rate.

NS_Age	NS_lx	NS_μ(x)	S_Age	S_lx	S_μ(x)
50	0.968	0.0023	50	0.937	0.0151
51	0.966	0.0024	51	0.923	0.0197
51	0.964	0.0047	53	0.905	0.0133
52	0.959	0.0047	54	0.893	0.0227
52	0.955	0.0072	56	0.873	0.0127
53	0.948	0.0169	57	0.862	0.0247
55	0.932	0.0049	58	0.841	0.0132
56	0.927	0.0074	59	0.830	0.0133
56	0.921	0.0049	59	0.819	0.0110
57	0.916	0.0175	60	0.810	0.0174
59	0.900	0.0076	61	0.796	0.0319
60	0.893	0.0025	62	0.771	0.0183
60	0.891	0.0102	63	0.757	0.0363
61	0.882	0.0052	64	0.730	0.0222
61	0.878	0.0078	65	0.714	0.0255
62	0.871	0.0105	66	0.696	0.0262
62	0.862	0.0079	66	0.678	0.0209
63	0.855	0.0107	67	0.664	0.0526
63	0.846	0.0081	68	0.630	0.0209
64	0.839	0.0054	69	0.617	0.0346
65	0.834	0.0082	69	0.596	0.0341
65	0.828	0.0083	70	0.576	0.0317
65	0.821	0.0083	71	0.558	0.0291
66	0.814	0.0112	71	0.542	0.0338
67	0.805	0.0056	72	0.524	0.0271
67	0.800	0.0143	73	0.510	0.0359
68	0.789	0.0087	73	0.492	0.0373
68	0.782	0.0087	74	0.474	0.0343
69	0.776	0.0177	74	0.458	0.0515
70	0.762	0.0180	75	0.435	0.0351
71	0.748	0.0465	76	0.420	0.0463
72	0.714	0.0257	76	0.401	0.0512
73	0.696	0.0231	77	0.381	0.0374
73	0.680	0.0236	77	0.367	0.0503
74	0.664	0.0207	78	0.349	0.0590
74	0.651	0.0211	78	0.329	0.0563
75	0.637	0.0252	79	0.311	0.0699
75	0.621	0.0221	79	0.290	0.1054
76	0.608	0.0342	80	0.261	0.0351
77	0.587	0.0394	80	0.252	0.0870
77	0.565	0.0494	81	0.231	0.0718
79	0.537	0.0744	82	0.215	0.0624
80	0.499	0.0465	82	0.202	0.0718
81	0.476	0.0588	83	0.188	0.0717
82	0.449	0.0518	84	0.175	0.0834
82	0.426	0.0603	84	0.161	0.1186
83	0.401	0.0642	85	0.143	0.0800
84	0.376	0.0621	86	0.132	0.1121
84	0.354	0.0459	86	0.118	0.1263
85	0.338	0.0840	87	0.104	0.1671
86	0.311	0.1926	88	0.088	0.1598
87	0.256	0.1527	89	0.075	0.2400
89	0.220	0.1927	89	0.059	0.2063
90	0.181	0.1054	90	0.048	0.1100
91	0.163	0.1178	91	0.043	0.0976
92	0.145	0.1158	92	0.039	0.1372
92	0.129	0.1112	92	0.034	0.2305
93	0.116	0.1252	93	0.027	0.4055
94	0.102	0.0000	94	0.018	0.1178
94	0.102	0.0000	95	0.016	0.1178

Table S2. Gompertzian hazard baseline estimation by smoking behaviors among British Male Doctors. Parameter estimates were obtained from Maximum Likelihood Estimation (MLE). C.I represents confidence intervals; Values are expressed to the nearest four decimal places. The absence of 95% C.I for γ in γ-Gompertz-Makeham model is due to low statistical power.

Models	Non-Smokers			*Smokers*		
		95% C.I			*95% C.I*	
	estimate	*lower*	*upper*	*estimate*	*lower*	*upper*
**Gompertz**						
a	*0.0035*	*0.0035*	*0.0036*	*0.0111*	*0.0109*	*0.0112*
b	*0.0871*	*0.0865*	*0.0877*	*0.0602*	*0.0596*	*0.0609*
**Gompertz Makeham**						
a	*0.0026*	*0.0024*	*0.0027*	*0.0027*	*0.0025*	*0.0029*
b	*0.0959*	*0.0947*	*0.0971*	*0.0986*	*0.0965*	*0.1007*
c	*0.0020*	*0.0017*	*0.0022*	*0.0133*	*0.0128*	*0.0138*
**γ-Gompertz Makeham**						
a	*0.0025*	*0.0024*	*0.0026*	*0.0027*	*0.0026*	*0.0029*
b	*0.0962*	*0.0950*	*0.0974*	*0.0986*	*0.0974*	*0.0999*
c	*0.0020*	*0.0018*	*0.0022*	*0.0133*	*0.0118*	*0.0148*
γ	*0.0014*	-	-	*0.0000*	-	-

Table S3. Gompertzian models and parameter estimates for British Male General Population. Data source: Human Mortality Database (HMD); Country: United Kingdom. Males born between year 1900-1909, and the parametric fit began from age 50 and onwards. C.I represents confidence intervals.

Models		*95% C.I*	
	estimate	*lower*	*upper*
**Gompertz**			
a	*0.0110*	*0.0110*	*0.0110*
b	*0.0808*	*0.0807*	*0.0809*
**Gompertz-Makeham**			
a	*0.0110*	*0.0110*	*0.0110*
b	*0.0808*	*0.0807*	*0.0809*
c	*0.0000*	*0.0000*	*0.0000*
**γ-Gompertz-Makeham**			
a	*0.0098*	*0.0098*	*0.0098*
b	*0.0923*	*0.0920*	*0.0925*
c	*0.0001*	*0.0001*	*0.0001*
γ	*0.1435*	*0.1402*	*0.1468*

Figure S1. Raw mortality rates from extracted survival curves on the absolute and semi-logarithmic scale; refer to Table S1. Non-smokers (blue); Smokers (red); General Population (green).

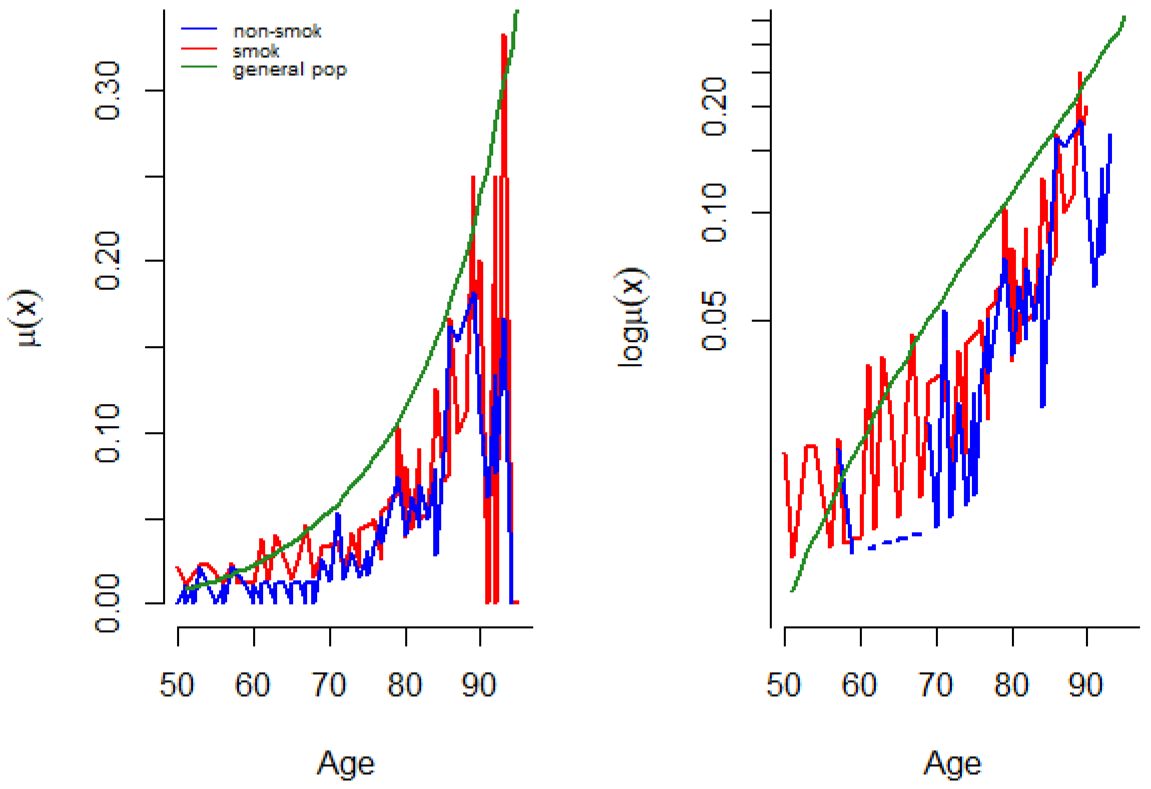

Figure S2. **Mortality rate for males in Britain at age 70 μ(70) between year 1920 to 2000.** Data source: HMD. A graphical representation of mortality improvement. The first evident reduction in mortality for μ(70) occurred in year 1970; *i.e.* males born after year 1900.

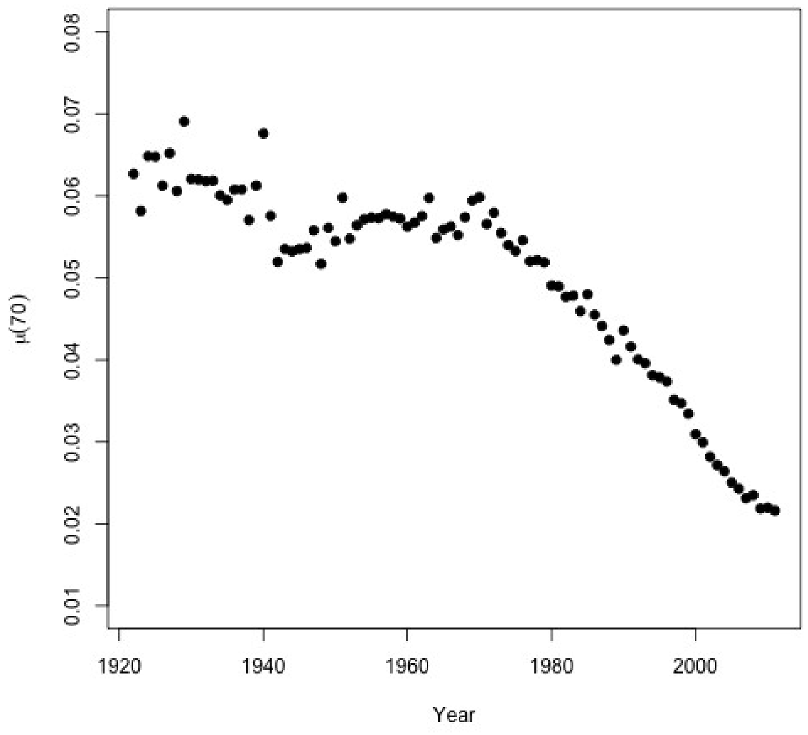

Supplement: Supplementary file 1 — Supplementary information [file 41598_2018_29081_MOESM1_ESM.pdf]
